# Supplementary material for: Genome-wide identification and expression profile analysis of CCH gene family in Populus
Source: PeerJ. 2017 Oct 27;5:e3962. doi: 10.7717/peerj.3962 (PMC5661435; doi:10.7717/peerj.3962)
Supplement: Table S1 [file peerj-05-3962-s001.docx]

**Table S1 List and description of** PtCCHs **and reference genes primers** **used for qRT-PCR analysis.**

| **Gene symbol** | **Locus name (POPTR)** | **Primers (5′-3′)** | **Product size (bp)** | **Annealing temp (°C)** |
| --- | --- | --- | --- | --- |
| *PtCCH1* | Potri.001G452400.1 | F: GTATGTTCTGGTGGTATGGTA  R: GAACTAGAAGGCAGAATTATTT | 139 | 47 |
| *PtCCH2* | Potri.001G468500.1 | F: GTTAAGCTGGCAAAGTCACTAAGAA  R: TAGAGCTGGCTGTGGATAACAA | 144 | 53 |
| *PtCCH3* | Potri.002G092200.1 | F: TTAAGGGTGTTAAATCTGTGGG  R: GATTCGGAGGTGCTTTCTTG | 195 | 53 |
| *PtCCH4* | Potri.004G056800.1 | F: AAAATCCTCATGCCTGCTCC  R: TGTAAGCCCAACCCAAAGAC | 182 | 52 |
| *PtCCH5* | Potri.005G003700.1 | F: AAAGGTGACCGTGACTGGGTTCGTG  R: TGATGGACTCGCTGATAGTGGCTGT | 200 | 51 |
| *PtCCH6* | Potri.005G079800.1 | F: AGTAGAGCAGCCTTAACACCC  R: ACGAAAGCCCAGACAGATAG | 241 | 53 |
| *PtCCH7* | Potri.005G110400.1 | F: GGGTACGTCCGAAATCCGCTTCA  R: TCGATCACAGCCCAGATTGACTA | 171 | 49 |
| *PtCCH8* | Potri.005G167000.1 | F: GAGGGCTGTGAACGTAAGA  R: GGGTTTGCCACTAAAGTGTA | 194 | 53 |
| *PtCCH10* | Potri.006G006100.4 | F: AACAGGTCAGCAGTATCGGG  R: ATTGCGGGGGAATTGTGG | 147 | 52 |
| *PtCCH11* | Potri.006G006100.5 | F: GCTGCAAGTGTCTCGAGGAT  R: GGGGTGAATGCAATAGTGGC | 110 | 54 |
| *PtCCH12* | Potri.006G024800.2 | F: ACGCCTTGTCTACTGTGGAGC  R: ACCCTTTTACAGTCACCTTTTGC | 147 | 53 |
| *PtCCH15* | Potri.010G114600.1 | F: TCACATCATCAAGTTTCCAA  R: GTCCCATATCAAATAAATACAGA | 130 | 46 |
| *PtCCH17* | Potri.011G065600.1 | F: AGCCCCCAAGATGACTATCAC  R: CTCTTCTCCCTGTCTTCCTCAC | 197 | 55 |
| *PtCCH19* | Potri.011G149500.1 | F: GTTCCAAGTGTGTAGCATCGC  R: TATATCCCCACGCCTGCAATC | 189 | 55 |
| *PtCCH20* | Potri.017G123400.1 | F: AAAGCAGGGTGGTGGTTAGTGGA  R: CTCTTGTCATAGGCACCGGAAAC | 145 | 60 |
| *PtUBQ7* | POPTR_0005s22060g | F: GGAACGGGTTGAGGAGAAAGAAG  R: GCAAGAACAAGATGAAGCACAGAGC | 135 | 55 |
| *PtCDC2* | POPTR_0004s14080g | F: ATTCCCCAAGTGGCCTTCTAAG  R: TATTCATGCTCCAAAGCACTCC | 137 | 57 |
