# Supplementary material for: Genome-wide identification and expression profile analysis of CCH gene family in Populus
Source: PeerJ. 2017 Oct 27;5:e3962. doi: 10.7717/peerj.3962 (PMC5661435; doi:10.7717/peerj.3962)
Supplement: Table S2 [file peerj-05-3962-s002.docx]

**Table S2**  **The amino acid identity of the 21 *PtCCH*s was aligned using BLAST in NCBI.**

| **Amino acid identity (%)** | | | | | | | | | | | | | | | | | | | | | |
| --- | --- | --- | --- | --- | --- | --- | --- | --- | --- | --- | --- | --- | --- | --- | --- | --- | --- | --- | --- | --- | --- |
| Protein name | PtCCH1 | PtCCH2 | PtCCH3 | PtCCH4 | PtCCH5 | PtCCH6 | PtCCH7 | PtCCH8 | PtCCH9 | PtCCH10 | PtCCH11 | PtCCH12 | PtCCH13 | PtCCH14 | PtCCH15 | PtCCH16 | PtCCH17 | PtCCH18 | PtCCH19 | PtCCH20 | PtCCH21 |
| PtCCH1 | 100 | 29 | 38 | 39 | 42 | 41 | 40 | 39 | 39 | 35 | 34 | 49 | 44 | 37 | 43 | 40 | 43 | 44 | 89 | 38 | 40 |
| PtCCH2 | - | 100 | 35 | 31 | 33 | 37 | 30 | 34 | 34 | 40 | 37 | 26 | 26 | 25 | 31 | 31 | 30 | 30 | 29 | 30 | 26 |
| PtCCH3 | - | - | 100 | 36 | 87 | 63 | 46 | 92 | 92 | 37 | 44 | 37 | 62 | 30 | 59 | 63 | 33 | 34 | 40 | 51 | 36 |
| PtCCH4 | - | - | - | 100 | 36 | 39 | 33 | 37 | 37 | 29 | 37 | 50 | 36 | 36 | 34 | 34 | 84 | 83 | 39 | 33 | 58 |
| PtCCH5 | - | - | - | - | 100 | 62 | 44 | 87 | 87 | 27 | 45 | 36 | 60 | 33 | 58 | 60 | 34 | 34 | 43 | 50 | 36 |
| PtCCH6 | - | - | - | - | - | 100 | 51 | 63 | 63 | 41 | 39 | 43 | 89 | 29 | 62 | 66 | 37 | 38 | 46 | 56 | 37 |
| PtCCH7 | - | - | - | - | - | - | 100 | 45 | 45 | 27 | 39 | 39 | 49 | 28 | 51 | 53 | 32 | 33 | 41 | 52 | 37 |
| PtCCH8 | - | - | - | - | - | - | - | 100 | 100 | 28 | 41 | 39 | 61 | 28 | 59 | 62 | 34 | 34 | 38 | 52 | 34 |
| PtCCH9 | - | - | - | - | - | - | - | - | 100 | 28 | 41 | 39 | 61 | 28 | 59 | 62 | 34 | 34 | 38 | 52 | 34 |
| PtCCH10 | - | - | - | - | - | - | - | - | - | 100 | 39 | 33 | 29 | 35 | 30 | 30 | 27 | 27 | 39 | 28 | 23 |
| PtCCH11 | - | - | - | - | - | - | - | - | - | - | 100 | 29 | 29 | 49 | 32 | 33 | 37 | 37 | 28 | 38 | 37 |
| PtCCH12 | - | - | - | - | - | - | - | - | - | - | - | 100 | 43 | 30 | 39 | 39 | 48 | 49 | 48 | 42 | 44 |
| PtCCH13 | - | - | - | - | - | - | - | - | - | - | - | - | 100 | 26 | 63 | 69 | 35 | 35 | 45 | 58 | 35 |
| PtCCH14 | - | - | - | - | - | - | - | - | - | - | - | - | - | 100 | 30 | 31 | 28 | 28 | 32 | 31 | 29 |
| PtCCH15 | - | - | - | - | - | - | - | - | - | - | - | - | - | - | 100 | 99 | 37 | 38 | 42 | 71 | 35 |
| PtCCH16 | - | - | - | - | - | - | - | - | - | - | - | - | - | - | - | 100 | 35 | 36 | 42 | 74 | 35 |
| PtCCH17 | - | - | - | - | - | - | - | - | - | - | - | - | - | - | - | - | 100 | 100 | 42 | 30 | 58 |
| PtCCH18 | - | - | - | - | - | - | - | - | - | - | - | - | - | - | - | - | - | 100 | 43 | 29 | 58 |
| PtCCH19 | - | - | - | - | - | - | - | - | - | - | - | - | - | - | - | - | - | - | 100 | 41 | 39 |
| PtCCH20 | - | - | - | - | - | - | - | - | - | - | - | - | - | - | - | - | - | - | - | 100 | 36 |
| PtCCH21 | - | - | - | - | - | - | - | - | - | - | - | - | - | - | - | - | - | - | - | - | 100 |
