# Supplementary material for: Genome-wide identification and expression profile analysis of CCH gene family in Populus
Source: PeerJ. 2017 Oct 27;5:e3962. doi: 10.7717/peerj.3962 (PMC5661435; doi:10.7717/peerj.3962)
Supplement: Table S3 [file peerj-05-3962-s003.docx]

**Table S3 46 of *CCH* genes identified from *Populus trichocarpa*, *Oryza sativa* and *Arabidopsis thaliana*.**

| **Organism** | **Gene name** | **Gene symbol** | **Gene description** | **Physical position (or clone number)** |
| --- | --- | --- | --- | --- |
| *Populus trichocarpa* | *PtCCH1* | Potri.001G452400.1 | Similar to copper chaperone (CCH)-related [ ortholog of At2g18196] | ChrI:48764846..48766902 |
|  | *PtCCH2* | Potri.001G468500.1 | Similar to hypothetical protein [ortholog of At3g20180] | ChrI:50097242..50098160 |
|  | *PtCCH3* | Potri.002G092200.1 | PTHR22814:SF84-Heavy-metal-associated domain-containing protein | ChrII:6570956..6572016 |
|  | *PtCCH4* | Potri.004G056800.1 | PTHR22814:SF107-F28N24.19 Protein | ChrIV:4523350..4524675 |
|  | *PtCCH5* | Potri.005G003700.1 | PTHR22814:SF84-Heavy-metal-associated domain-containing protein | ChrV:226035..226652 |
|  | *PtCCH6* | Potri.005G079800.1 | Similar to copper chaperone (CCH)-related | ChrV:5867660..5868994 |
|  | *PtCCH7* | Potri.005G110400.1 | PTHR22814:SF155-Heavy-metal-associated isoprenylated plant protein 26 | ChrV:8497273..8498140 |
|  | *PtCCH8* | Potri.005G167000.1 | PTHR22814:SF84-Heavy-metal-associated domain-containing protein | ChrV:17596661..17597639 |
|  | *PtCCH9* | Potri.005G169700.1 | PTHR22814:SF84-Heavy-metal-associated domain-containing protein | ChrV:18274591..18275572 |
|  | *PtCCH10* | Potri.006G006100.4 | PTHR22814//PTHR22814:SF94-Copper transport protein ATOX1-related | ChrVI:415310..419144 |
|  | *PtCCH11* | Potri.006G006100.5 | PTHR22814//PTHR22814:SF94-Copper transport protein ATOX1-related | ChrVI:416730..419039 |
|  | *PtCCH12* | Potri.006G024800.2 | PTHR22814:SF85-Metal ion binding protein | ChrVI:1722458..1723578 |
|  | *PtCCH13* | Potri.007G087300.1 | PTHR22814//PTHR22814:SF126-Copper transport protein ATOX1-related | ChrVII:11343882..11345554 |
|  | *PtCCH14* | Potri.010G015300.1 | Copper-binding family protein | ChrX:1921667..1922272 |
|  | *PtCCH15* | Potri.010G114600.1 | Similar to copper chaperone (CCH)-related | ChrX:13289528..13291583 |
|  | *PtCCH16* | Potri.010G114600.2 | Similar to copper chaperone (CCH)-related | ChrX:13289596..13291583 |
|  | *PtCCH17* | Potri.011G065600.1 | PTHR22814:SF107-F28N24.19 Protein | ChrXI:6016497..6017737 |
|  | *PtCCH18* | Potri.011G065600.2 | PTHR22814:SF107-F28N24.19 Protein | ChrXI:6016577..6017733 |
|  | *PtCCH19* | Potri.011G149500.1 | PTHR22814:SF136-Heavy-metal-associated domain-containing protein-related | ChrXI:16795622..16798712 |
|  | *PtCCH20* | Potri.017G123400.1 | Similar to copper chaperone (CCH)-related | ChrXVII:13590414..13592054 |
|  | *PtCCH21* | Potri.019G106500.1 | Copper-binding family protein | ChrXIX:13509997..13510615 |
| *Oryza sativa* | *OsCCH1* | LOC_Os01g20830.1 | Heavy metal-associated domain containing protein, expressed | ChrI:11610848..11612708 |
|  | *OsCCH2* | LOC_Os01g32330.1 | Heavy metal-associated domain containing protein, expressed | ChrI:17740974..17742308 |
|  | *OsCCH3* | LOC_Os01g55320.1 | Heavy metal-associated domain containing protein, expressed | ChrI:31840530..31841097 |
|  | *OsCCH4* | LOC_Os02g30650.2 | Heavy metal-associated domain containing protein, expressed | ChrII:18252570..18256018 |
|  | *OsCCH5* | LOC_Os02g32814.1 | Heavy metal-associated domain containing protein, expressed | ChrII:19472211..19486248 |
|  | *OsCCH6* | LOC_Os03g02860.1 | Heavy metal-associated domain containing protein, expressed | ChrIII:1124956..1127202 |
|  | *OsCCH7* | LOC_Os03g06080.1 | Heavy metal-associated domain containing protein, expressed | ChrIII:3046790..3047842 |
|  | *OsCCH8* | LOC_Os03g26650.1 | Heavy metal-associated domain containing protein, expressed | ChrIII:15224017..15225611 |
|  | *OsCCH9* | LOC_Os03g26650.2 | Heavy metal-associated domain containing protein, expressed | ChrIII:15224017..15225611 |
|  | *OsCCH10* | LOC_Os08g31140.1 | Heavy metal-associated domain containing protein, expressed | ChrVIII:19244362..19245760 |
|  | *OsCCH11* | LOC_Os09g20000.1 | Heavy metal-associated domain containing protein, expressed | ChrIX:11976355..11978024 |
|  | *OsCCH12* | LOC_Os10g39210.1 | Heavy metal-associated domain containing protein, expressed | ChrX:20935118..20935789 |
| *Arabidopsis thaliana* | *AtCCH1* | AT1G06330.1 | Heavy metal transport/detoxification superfamily protein | ChrI:1931671..1932266 |
|  | *AtCCH2* | AT1G29100.1 | Heavy metal transport/detoxification superfamily protein | ChrI:10169084..10169619 |
|  | *AtCCH3* | AT1G71050.1 | Heavy metal transport/detoxification superfamily protein | ChrI:26803153..26804292 |
|  | *AtCCH4* | AT2G18196.1 | Heavy metal transport/detoxification superfamily protein | ChrII:7920508..7922326 |
|  | *AtCCH5* | AT3G21490.1 | Heavy metal transport/detoxification superfamily protein | ChrIII:7573065..7573669 |
|  | *AtCCH6* | AT3G56240.1 | Copper chaperone | ChrIII:20863257..20864544 |
|  | *AtCCH7* | AT3G56891.1 | Heavy metal transport/detoxification superfamily protein | ChrIII:21064199..21064922 |
|  | *AtCCH8* | AT4G10465.1 | Heavy metal transport/detoxification superfamily protein | ChrIV:6473547..6475434 |
|  | *AtCCH9* | AT4G38580.1 | Farnesylated protein 6 | ChrIV:18034431..18035889 |
|  | *AtCCH10* | AT4G39700.1 | Heavy metal transport/detoxification superfamily protein | ChrIV:18424265..18424906 |
|  | *AtCCH11* | AT5G17450.1 | Heavy metal transport/detoxification superfamily protein | ChrV:5755322..5756888 |
|  | *AtCCH12* | AT5G17450.2 | Heavy metal transport/detoxification superfamily protein | ChrV:5755348..5756878 |
|  | *AtCCH13* | AT5G66110.1 | Heavy metal transport/detoxification superfamily protein | ChrV:26430202..26430912 |
