# Supplementary material for: Genome-wide identification and expression profile analysis of CCH gene family in Populus
Source: PeerJ. 2017 Oct 27;5:e3962. doi: 10.7717/peerj.3962 (PMC5661435; doi:10.7717/peerj.3962)
Supplement: Table S4 [file peerj-05-3962-s004.docx]

**Table S4**  **Motif sequences of CCH proteins identified in *P.* *trichocarpa* by MEME tools.**

| **Motif** | **Width** | **E-value** | **Best possible match** |
| --- | --- | --- | --- |
| 1 | 30 | 1.5E-131 | GVKSVEVDMKQQKVTVTGYVDPNKVLKAVR |
| 2 | 22 | 2.2E-6 | TIFDDNYTNMFSDENPNACSIM |
| 3 | 22 | 1.7E-114 | LKVRMDCEGCERKVKNAISKLK |
| 4 | 12 | 3.8E-96 | STGKRAEFWPYV |
| 5 | 31 | 9.6E-108 | PYTLVAYPYVSQAYDKKAPPGYVRNVPVTAT |
| 6 | 22 | 2.5E-99 | AGSYNYYSHLYNNAKKGRKRKP |
| 7 | 19 | 2.4E-71 | NNPESYNFNQQYYYQKQHH |
| 8 | 31 | 7.1E-114 | ITYSYFYFREDHVRIKNIRHNMPKGRPLSLQ |
| 9 | 16 | 7.1E-114 | YFTSANNYFKDTTSEF |
| 10 | 9 | 3.8E-21 | MATILERAF |
