# Supplementary material for: Genome-wide identification and expression profile analysis of CCH gene family in Populus
Source: PeerJ. 2017 Oct 27;5:e3962. doi: 10.7717/peerj.3962 (PMC5661435; doi:10.7717/peerj.3962)
Supplement: Table S5 — ∗ represents statistical significant differences ( P < 0.05) using t test. [file peerj-05-3962-s005.docx]

**Table S5 The effect of copper stress on the expression profiles of *PnCCH* genes in different tissues tested by qRT-PCR.**

|  | **Young leaves** | | | | | | **Mature leaves** | | | | | |
| --- | --- | --- | --- | --- | --- | --- | --- | --- | --- | --- | --- | --- |
| **Gene** | **0 μmol/L Cu** | | | **10 μmol/L Cu** | | | **0 μmol/L Cu** | | | **10 μmol/L Cu** | | |
|  | 3 h | 12 h | 72 h | 3 h | 12 h | 72 h | 3 h | 12 h | 72 h | 3 h | 12 h | 72 h |
| PnCCH1 | -0.94±0.08* | -0.84±0.07* | 0.37±0.16* | -0.36±0.36 | 0.23±0.15 | -1.05±0.02* | -0.23±0.10* | 0.05±0.06 | 0.12±0.10 | -0.60±0.14* | -0.85±0.09* | 0.65±0.07* |
| PnCCH2 | -1.34±0.07* | -0.66±0.03* | 1.05±0.19* | -0.79±0.37* | -0.43±0.26 | -1.67±0.09* | -0.06±0.04 | -0.11±0.02* | 1.04±0.05* | 0.80±0.04* | -0.38±0.01* | 1.37±0.26* |
| PnCCH3 | -2.72±0.17* | 1.14±0.33* | 3.31±0.30* | -0.18±0.15 | 3.27±0.13* | 5.41±0.19* | -2.36±0.55* | 3.41±0.20* | 0.12±0.78 | -1.61±0.10* | 1.44±1.22 | 0.38±0.46 |
| PnCCH4 | -1.59±1.21 | -0.11±0.18 | 0.05±0.15 | -2.25±0.12* | 1.54±0.25* | -1.32±0.46* | 0.23±0.16 | 0.18±0.06* | -1.16±0.09* | -1.23±0.07* | -2.19±0.18* | -0.09±0.25 |
| PnCCH5 | -2.52±0.51* | 2.03±0.12* | -0.99±0.12* | -2.20±0.23* | 1.16±0.18* | 0.81±0.19 | 0.51±0.04* | -3.59±2.96 | -3.96±0.49* | -0.92±0.09* | -0.15±0.47 | -2.53±0.30* |
| PnCCH6 | -0.83±0.23 | -0.40±0.07 | -0.76±0.50* | -0.01±0.27 | 0.90±0.04* | -0.55±0.11* | -0.02±0.14 | 1.04±0.16* | 3.26±0.15* | 0.90±0.05* | 0.40±0.10* | 2.33±0.28* |
| PnCCH7 | 1.40±0.77 | 0.42±0.26 | 0.90±0.37 | 0.36±0.37 | 0.11±0.47 | 5.77±0.12* | -0.62±0.54 | 4.63±0.12* | 1.34±0.49 | -0.50±0.20 | 1.87±0.13* | 0.84±0.20* |
| PnCCH8 | -1.59±0.84* | 0.46±0.34 | -0.56±0.26* | -2.56±0.29* | 7.11±0.13* | 5.14±0.11* | 1.21±0.14* | 2.27±0.18* | 6.19±0.16* | 1.28±0.27* | 4.48±0.15* | 5.52±0.13* |
| PnCCH10 | -2.10±0.14* | -0.44±0.15* | -1.27±0.08* | -1.45±0.12* | 1.43±0.15* | -3.50±0.10* | -2.25±0.03* | -0.50±0.14* | 0.73±0.25* | -1.16±0.02* | -2.54±0.01* | 0.19±0.19 |
| PnCCH11 | -2.00±0.35* | 0.79±0.09 | -1.55±0.10* | -0.28±0.11 | 0.06±0.13* | -3.18±0.11* | -3.25±0.19* | 1.41±0.05* | 0.99±0.46 | -4.85±0.09* | 0.07±0.39* | -0.27±0.16* |
| PnCCH12 | -2.57±0.07* | -1.65±0.12* | 0.91±0.92 | -1.32±0.09* | -1.58±0.04* | -1.66±0.34* | -0.98±0.06* | -1.06±0.04* | -0.43±0.01 | -0.57±0.06* | -2.22±0.03* | -0.51±0.11 |
| PnCCH15 | -2.38±0.15* | -1.01±0.52 | -1.94±0.67* | -1.57±0.13* | 2.22±0.31* | -0.213±0.07 | -1.28±0.14* | -0.04±0.14 | 3.21±0.05* | -0.45±0.23* | -1.55±0.15* | 2.47±0.18* |
| PnCCH17 | -1.02±0.10* | -0.43±0.12* | -0.62±0.87 | -0.49±0.16* | 0.18±0.25 | -1.92±0.08* | 0.11±0.44 | 0.09±0.03 | -1.25±0.30* | -0.43±0.06* | -1.59±0.13* | -0.85±0.04* |
| PnCCH19 | -1.10±0.15* | -1.31±0.15* | 0.78±0.53 | 0.92±0.37* | 2.02±0.29* | 0.16±0.35 | -0.32±0.14* | 0.14±0.20 | -1.20±0.19* | -0.14±0.17 | -1.17±0.05* | -0.76±0.33* |
| PnCCH20 | -3.34±0.46* | 1.34±0.24* | 1.03±0.59 | -1.75±0.45* | 0.36±0.79 | 0.60±0.12 | -0.23±0.17 | 1.16±0.15* | 2.17±0.42* | 0.76±0.21* | 0.40±0.38 | -2.41±0.13* |
|  | Stems | | | | | | Roots | | | | | |
| gene | 0 μmol/L Cu | | | 10 μmol/L Cu | | | 0 μmol/L Cu | | | 10 μmol/L Cu | | |
|  | 3 h | 12 h | 72 h | 3 h | 12 h | 72 h | 3 h | 12 h | 72 h | 3 h | 12 h | 72 h |
| PnCCH1 | -1.62±0.33* | 1.39±0.14* | 1.25±0.33* | -0.49±0.53 | 1.44±0.22* | -1.00±0.57 | 3.05±0.17* | -0.05±0.34 | -1.06±0.17* | 2.99±0.01* | 0.05±0.17 | -4.22±0.71* |
| PnCCH2 | -1.43±0.45* | -1.37±1.20 | 0.51±0.17* | -0.69±0.53 | -0.67±0.19 | -0.40±0.14* | 1.44±0.10* | 1.26±0.19 | -1.93±0.14 | 1.53±0.99 | 3.82±0.08 | 1.48±0.23 |
| PnCCH3 | -0.03±0.31 | 0.51±0.20 | 1.11±0.04* | -2.49±0.53* | 4.55±0.12* | 4.15±0.14* | -0.34±0.19 | 1.08±0.62 | 0.35±0.05 | -1.21±0.33* | 2.55±0.08* | -0.19±0.26 |
| PnCCH4 | -0.57±0.10 | 4.66±0.40* | 1.53±0.64* | 1.56±0.33* | 1.17±0.83 | 1.99±0.14* | 0.46±0.18* | 1.00±0.16* | -1.66±0.22* | 2.59±0.12* | 6.21±0.26* | -2.15±0.24* |
| PnCCH5 | 2.14±0.29* | 0.39±0.49 | 2.35±0.20* | 2.38±0.28* | 2.05±0.10* | 2.45±0.27* | 0.98±0.34* | 1.83±0.33* | 1.51±0.20* | 0.46±0.18 | 2.76±0.33* | 2.27±0.16* |
| PnCCH6 | 0.04±0.50 | -0.54±0.05 | -0.02±0.38 | 0.14±0.46 | -3.10±0.31* | 1.87±0.30* | 0.04±0.12 | 0.45±0.32 | 1.78±0.43* | 0.29±0.30 | 0.28±0.04 | -1.53±0.39* |
| PnCCH7 | 0.88±0.09* | -0.81±0.69 | 1.83±0.87 | 0.84±0.32* | 1.00±0.24* | 3.66±0.44* | 1.46±0.23* | -0.54±0.35 | -0.48±0.16 | 0.35±0.38 | 3.05±0.38* | -0.03±0.43 |
| PnCCH8 | 0.03±0.45 | -0.98±0.53* | 0.70±0.46* | 1.58±0.31* | 2.18±0.20* | 5.64±0.09* | 0.69±0.25 | 0.74±0.26* | 0.15±0.22 | 0.63±0.34 | 4.37±0.10* | 0.84±0.09* |
| PnCCH10 | 2.41±0.17* | -0.85±0.27* | 1.82±0.09* | 2.01±0.28* | -2.82±0.06* | -0.74±0.28* | 0.13±0.36 | 1.53±0.09* | -0.17±0.21 | 0.29±0.73 | 2.05±0.44* | -0.51±0.18 |
| PnCCH11 | -2.00±0.22 | -0.10±0.52* | -0.41±1.34 | -2.30±0.21 | 0.06±0.41* | -1.54±0.21* | 0.68±0.11 | 0.80±0.20 | 0.35±0.30 | 1.31±0.36 | 0.67±0.25* | 1.47±0.36* |
| PnCCH12 | 2.11±0.90* | -0.59±0.12 | -2.83±0.23* | -1.12±0.35* | -0.27±0.20 | -4.17±0.50* | 1.00±0.28 | -4.14±0.12* | 0.31±0.80 | 1.85±0.14 | -4.58±0.55* | -0.37±0.34 |
| PnCCH15 | -2.23±0.81* | -1.21±0.68* | 1.66±1.18 | 1.08±0.11* | -2.10±0.90* | 1.11±0.57 | 0.08±0.35 | -3.27±0.53* | -0.59±0.53 | 0.93±0.16* | -1.05±0.21* | -2.06±0.62* |
| PnCCH17 | 0.74±0.06* | 0.53±0.13* | 0.69±0.27* | 1.55±0.57* | 1.66±0.24* | 0.74±0.23* | 0.22±0.08 | -2.46±0.23* | -0.94±0.11* | 5.48±0.13* | 4.81±0.21* | -0.81±0.15* |
| PnCCH19 | 0.10±0.13 | 0.22±0.09* | 0.47±0.01* | 0.12±0.44 | 0.09±0.17 | -1.76±0.10* | -0.67±0.05* | -3.35±0.04* | -1.34±0.25* | 1.70±0.16* | -3.07±0.35* | -3.46±1.03* |
| PnCCH20 | -1.14±0.27* | -0.14±0.05 | -0.31±0.40 | -1.63±0.24* | -1.48±0.22* | -3.96±0.54* | 0.02±0.21 | -0.34±0.28 | -0.03±0.52 | 0.25±0.16 | -4.96±0.23* | -1.52±0.14* |

The 2^−ΔΔCT^ method was used to analyze the expression levels of *PnCCHs* in different tissues upon to copper stress conditions. In this experiment, 0 μM CuSO_4_ is deficiency copper condition, 10 μM CuSO_4_ is excessive copper condition, and 0.5 μM CuSO_4_ is control copper condition. The statistical significant differences of the expression levels were analyzed using t test (*P*<0.05). All the values under copper stress conditions were compared with the corresponding values under control copper condition. Results are the mean ± SD of three replicates, and * represents statistical significant differences (*P* < 0.05) using t test.
