# Supplementary figures and images for: Genome-wide identification and expression profile analysis of CCH gene family in Populus
Source: PeerJ. 2017 Oct 27;5:e3962. doi: 10.7717/peerj.3962 (PMC5661435; doi:10.7717/peerj.3962)

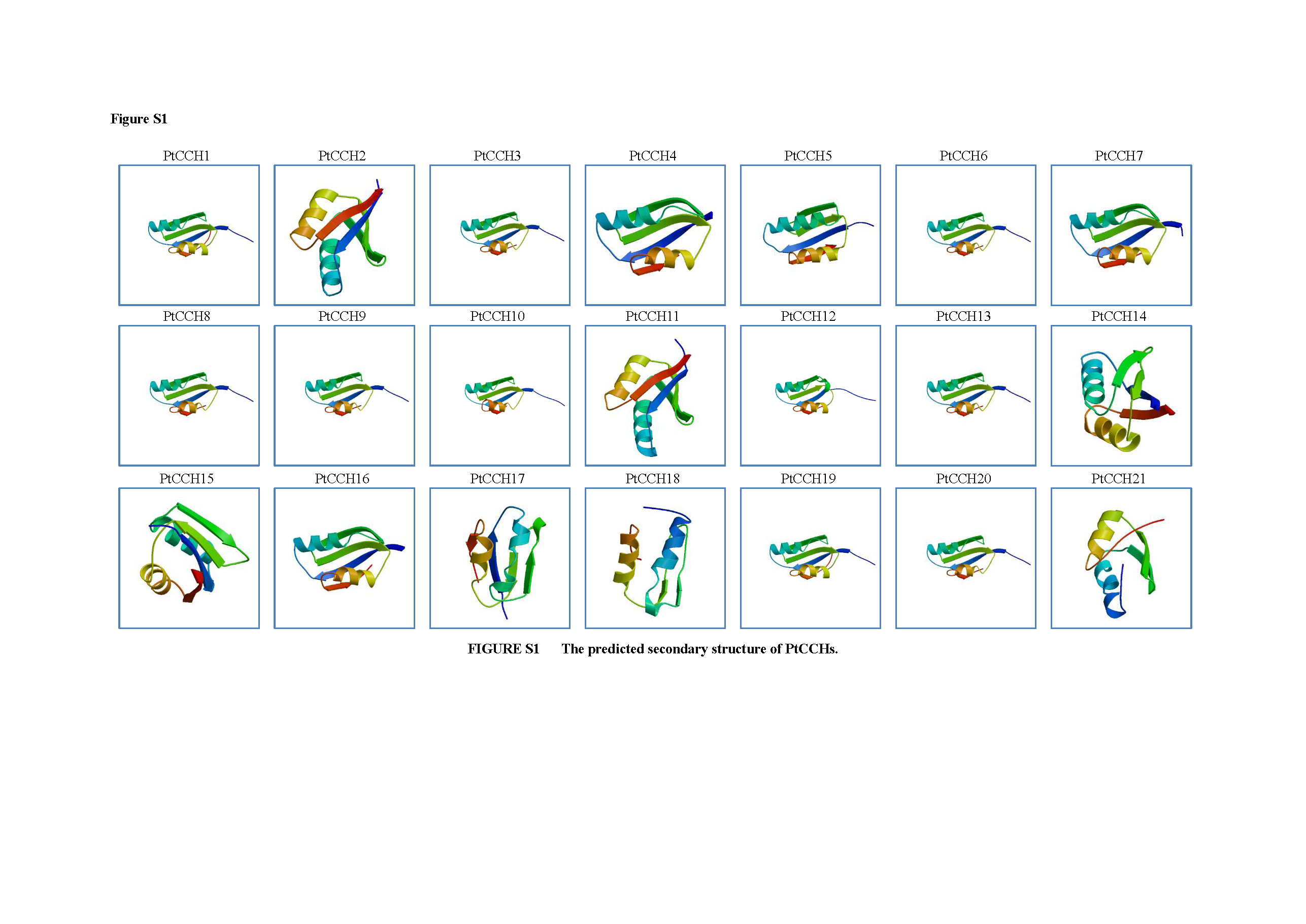

Supplement: Figure S1 [file peerj-05-3962-s006.png]
