# Supplementary material for: Genome-wide identification and expression profile analysis of CCH gene family in Populus
Source: PeerJ. 2017 Oct 27;5:e3962. doi: 10.7717/peerj.3962 (PMC5661435; doi:10.7717/peerj.3962)
Supplement: Supplemental Information 1 [file peerj-05-3962-s007.doc]

**1. PnCCH1**

nucleotide sequence:

ATGGCTACCATCTTAGAGCGAGCATTCAAGTCTTTCTTATCTAATTCCATTGCTTACTGATCTTACTTTAAATTTACTTTCGAGAAGACCACACGAGGTTCGAAAATATCAAATACAACATGCCTAAGGGGAGACCACTTTCTTTGCAGACTGTGGAGCTCAAAGTCAGGATGTGCTGCACTGGCTGTGAAAGAGTTGTCAAAAATGCCATTTACAAGCTTAAAGGTATTGATTCAGTGGAGGTGGACTTGGAGATGGAAAAGGTAACTGTAGTGGGATATGTTGATCGAAACAAGGTGCTGAAGGCGGTGAGGAGGGCAGGAAAGAGAGCAGAATTCTGGCCCTATCCGAATCCGCCATTGTACTTCACATCAGCTAATCACTATTTCAAGGACACAACTAGTGAGTTCAAAGAGAGTTACAACTATTACAAGCATGGCTACAACCTTGCAGACAGGCATGGGACCATCCCGGTGAGCCACCGGGGAGACGACAAGGTCAGCAACATGTTTAACGATGACAACGTTAATGCCTGCTGTCTCATGTAG

amino acid sequence:

MATILERAFKSFLSNSIAY-SYFKFTFEKTTRGSKISNTTCLRGDHFLCRLWSSKSGCAALAVKELSKMPFTSLKVLIQWRWTWRWKR-L-WDMLIETRC-RR-GGQEREQNSGPIRIRHCTSHQLITISRTQLVSSKRVTTITSMATTLQTGMGPSR-ATGETTRSATCLTMTTLMPAVSC

**2. PnCCH2**

nucleotide sequence:

ATGAAGCAAAAAATAGTGATCAAAGTGCATATGCATTGCGAGAAATGTAGAACCAAAGCCAAGAAGATTGCTGCCACGGCATGTGGTGTAACTTCGGTGGCGCTGGAAGCAGCCAAAGATCAGATAGTGGTGATCGGAGAGGAAGTTGATTCGGTTAAGCTGGCAAAGTCACTAAGAAAAAAGGTTGGCCATGCCGTTTTGATGAGCGTGCAAGAAGAGAAAGAGAAAGCGAAAGATAAAGATGAGAAGAAAGGAGATGATAAAAAACCAACTCTATATTGTTATCCACAGCCTCTACCACATGCAGTGGTTTGTGAATCAAACCCTGACAATTGCACCGTATTGTGA

amino acid sequence:

MKQKIVIKVHMHCEKCRTKAKKIAATACGVTSVALEAAKDQIVVIGEEVDSVKLAKSLRKKVGHAVLMSVQEEKEKAKDKDEKKGDDKKPTLYCYPQPLPHAVVCESNPDNCTVL

**3. PnCCH3**

nucleotide sequence:

ATGGGAGTTGCAGGAACTTTGGAGTACTTCTCTGATTTACTAAGCAATGTCAAGAAGGGCAAGAAAAGGAAGCAAATGCAAACTGTAGCACTCAAAGTCAGGATGGACTGCGAGGGCTGTGAACGTAAGATCAAGAGTGTCCTCTCCGGAGTTAAAGGTGTTAAATCTGTGGACGTCGACATGAAGCAACAAAAGGTGACTGTGACTGGGTACGTAGAGCCAAAGAAAGTGTTGAAGGCAGCTCAATCGACAAAGAAGAAGGTGGAGATGTGGCCTTATGTGCCATACACTTTAGTGGCAAACCCCTATGTTTCACAGGCTTATGACAAGAAAGCACCTGCTAATCATGTCAGAGCCGTTCCGGTCACCGCCACCATCAGCGAGACCACCATGGACGACAACTACACCAACATGTTTAGTGATGAGAACCCCAATGCCTGCTCCATCATGTAA

amino acid sequence:

MGVAGTLEYFSDLLSNVKKGKKRKQMQTVALKVRMDCEGCERKIKSVLSGVKGVKSVDVDMKQQKVTVTGYVEPKKVLKAAQSTKKKVEMWPYVPYTLVANPYVSQAYDKKAPANHVRAVPVTATISETTMDDNYTNMFSDENPNACSIM

**4. PnCCH4**

nucleotide sequence:

ATGACTATCACAGAGATGAAAGTCTATATGGATTGTGCTGGCTGCGAGACCAAGATAAGGAAGGCTATTCAAAAACTAGATGGAGTGGATGATATCGATATAGACATATATATGCAAAAAGTAACAGTTATGGGATGGGCAGACCAGAGAAAAGTTCTTAAAGCAGTGAGGAAGACAGGAAGAAGAGCTGAGCTATGGCCATACCCATACAATCCTGAATCCTATAACTTCAACCAACAGTACTATTATCAGCAGCAGCATGAACAAGAAACAGTTACTTACTATGAAAAGAAGCCTACCGCTTCATACAACTACGACAAGCATGGCTACAATGAAGAAGAGTTTGGTTACTATCAAAAGCCAGCTTATGCCACCATTGTTGATGAAGAAGCTAGTGCCATCTTCAGTGATGAAAATCCTCATGCCTGCTCCATCATGTAA

amino acid sequence:

MTITEMKVYMDCAGCETKIRKAIQKLDGVDDIDIDIYMQKVTVMGWADQRKVLKAVRKTGRRAELWPYPYNPESYNFNQQYYYQQQHEQETVTYYEKKPTASYNYDKHGYNEEEFGYYQKPAYATIVDEEASAIFSDENPHACSIM

**5. PnCCH5**

nucleotide sequence:

ATGGGAGTTGCCGGAACTCTGGAGTATTTCTCTGATTTACTAAGCAATGTCAAGAAAGGCAAGAAAAAGAAGCTGATGCAAACCGTAGCTCTCAAAGTCAGGATGGACTGCCAAGTCTGTGAACGTAAGGTCAAGAGTGTCCTCTACGGGGTTGATGGTGTTAAATCCGTGAAAGTAGACATGAAGCAACAAAAGGTGACCGTGACTGGGTTCGTGGAGCCAGAGAAAGTGTTGAAGGCAGCTCAATCAACAAAGAAGAAGGTAGAGCTGTGGCCTTATGTCCCATACTTTTTAGTGGCACACCCTTATGTTTCACAGGCTTATGACAATAAAGCACCTCCGAATCATGTTAGAGCAGTTCCGGTCACAGCCACTATCAGCGAGTCCATCATTGACGACTACTACATCAACATGTTTAGTGATGAGAACCCTAATGCCTGCTCCATTATGTAA

amino acid sequence:

MGVAGTLEYFSDLLSNVKKGKKKKLMQTVALKVRMDCQVCERKVKSVLYGVDGVKSVKVDMKQQKVTVTGFVEPEKVLKAAQSTKKKVELWPYVPYFLVAHPYVSQAYDNKAPPNHVRAVPVTATISESIIDDYYINMFSDENPNACSIM

**6. PnCCH6**

nucleotide sequence:

ATGGGAGTTAGTGGCACTTTGGAGTATTTATCTGACTTGGTGGGAAGTGGAGGCCATAAACACAAGAAGAAGAAGCAGTTACAGACTGTTGAGCTTAAGGTCAGGATGGACTGTGATGGCTGTGAACTTAAGGTCAAGAAGGCCATTTCTTCATTGAGTGGAGTTAAAAAGGTGGAGATAAACAGAAAACAACAAAGGGTGACTGTTACAGGATATGTTGATTCAAGTAAGGTGTTGAAGAAGGCAAAGTCAACAGGGAAAAAGGCAGAGATTTGGCCGTATGTTCCTTACAATTTAGTGGCTCAACCTTACGCTGTTCAGGCTTATGACAAGAAGGCTCCTCCTGGTTATGTCAGGAATGTAGAAAACACGGTCACCACAGGCACCGTGACCAGATATGAGGACCCCTACACCTCCATGTTCAGTGACGACAACCCAAATGCTTGCTCTATCATGTAA

amino acid sequence:

MGVSGTLEYLSDLVGSGGHKHKKKKQLQTVELKVRMDCDGCELKVKKAISSLSGVKKVEINRKQQRVTVTGYVDSSKVLKKAKSTGKKAEIWPYVPYNLVAQPYAVQAYDKKAPPGYVRNVENTVTTGTVTRYEDPYTSMFSDDNPNACSIM

**7. PnCCH7**

nucleotide sequence:

ATGGGGTGTCTGGATCGCGTCTCCGAACTCTGTCACTGGCCCCATGATAGCACGAGACTCAGAAAACGCGAGCCATTAGAGACAGTGGAGATTAAAGTGAAAATGGACTGTGAAGGGTGTGAGACAAAGGTGAGAAACTCAGTGACAGGGATGAAAGGAGTGATCCAAGTAGAAGTGGACCGCAAACTACAGAAACTGACGGTGACCGGATACGTGGACCCGGACGAGGTCTTGCACCGTGTGAGGTATAGAACGGGAAAGAAAGCTGAGTTCTGGCCGTATGTGCCGGCTGAGGTAGTCCCTCTCCCATACTCCGCTGGAGTGTATGATAAGAAAGCCCCACCTGGGTACGTCCGAAATCCGCTTCAGCTCGAGGACCCACCGGCCTCCAGTTCTTTCGAGGTGAAGACCACTACAGCTTTCAGTGATGATAATCCAAATGCTTGTGTGATAATGTGA

amino acid sequence:

MGCLDRVSELCHWPHDSTRLRKREPLETVEIKVKMDCEGCETKVRNSVTGMKGVIQVEVDRKLQKLTVTGYVDPDEVLHRVRYRTGKKAEFWPYVPAEVVPLPYSAGVYDKKAPPGYVRNPLQLEDPPASSSFEVKTTTAFSDDNPNACVIM

**8. PnCCH8**

nucleotide sequence:

ATGGGAGTTGCAGGAACTTTGGAGTACTTCTCTGATTTACTAAGCAATGTCAAGAAGGGCAAGAAAAGGAAGCAAATGCAAACTGTAGCACTCAAAGTCAGGATGGACTGCGAGGGCTGTGAACGTAAGATCAAGAGTGTCCTCTCCGGAGTTAAAGGTGTTAAATCTGTGGACGTCGACATGAAGCAACAAAAGGTGACTGTGACTGGTTACGCAGAGCCAAAGAAAGTGTTGAAGGCAGCTCAATCGACAAAGAAGAAGGTGGAGATGTGGCCTTATGTGCCATACACTTTAGTGGCAAACCCCTATGTTTCACAGGCTTATGACAAGAAAGCACCTGCTAATCATGTCAGAGCCGTTCCGGTCACCGCCACCATCAGCGAGACCACCATGGACGACAACTACACCAACATGTTTAGTGATGAGAACCCCAATGCCTGTTCCATCATGTAG

amino acid sequence:

MGVAGTLEYFSDLLSNVKKGKKRKQMQTVALKVRMDCEGCERKIKSVLSGVKGVKSVDVDMKQQKVTVTGYAEPKKVLKAAQSTKKKVEMWPYVPYTLVANPYVSQAYDKKAPANHVRAVPVTATISETTMDDNYTNMFSDENPNACSIM

**9. PnCCH10**

nucleotide sequence:

ATGAGCTGCAAGTGTCTCGAGGATGGTGGTCTTAATGGAATTTGGTGGTTTCTGTCTCACTCTTGTTGGCCTCCTCCCGTTGTAACTGTAGTCTTGAACGTTAGAATGCATTGCGAAGCATGTGCTCAAGTGCTACAAAAGCGAGTTCGAAAGATCCAAGGTGTAGAGTCAGTAGAAACAAACCTAGCCAATGATCAAGTAATAGTAAAAGGGGTGGTTGATCCATCAAAGCTGGTGGATGATGTGTACAAGAAGACTAGAAAACAAGCTTCTATAGTGAAAGATGAAGAAAAGAAGGAAGAAGAGAAGAAAGAAGAGAAAAAGGAAGAAAAAGAAGGAGAAAAGAAAGATGGAGAAGAAGGGAAGGCAGAGGATGACAAGAACCTAGATATCAAGAGAAGTGAATATTGGCCATCAAAGTACTACTCTGAGTTTGCTTATGCTCCTCAGATTTTCAGTGATGAAAACCCTAATGCTTGCTCTGTTATGTAA

amino acid sequence:

MSCKCLEDGGLNGIWWFLSHSCWPPPVVTVVLNVRMHCEACAQVLQKRVRKIQGVESVETNLANDQVIVKGVVDPSKLVDDVYKKTRKQASIVKDEEKKEEEKKEEKKEEKEGEKKDGEEGKAEDDKNLDIKRSEYWPSKYYSEFAYAPQIFSDENPNACSVM

**10. PnCCH11**

nucleotide sequence:

ATGGGTGAAGAGAAAAAACAGGAAGAAAAGAAAGAAGAGGCCAAGGAAGAAGAGAAGAAGGAAGAAAAGAAAGAAGAAGAGCCTCCAGAGATTGTGCTCAAGGTTGATATGCATTGTGAAGCTTGTGCCAGGAAAGTTGCAAGAGCTTTGAAAGGATTTGAAGGAGTGGAGCAAGTAAGCACAGATAGCAAAGCAAGCAAGGTGGTGGTGAAAGGCAAAGCAGCAGACCCATCAAAGGTATGTGAGAGGCTGCAAAAGAAAAGTGGCCGGAAAGTGGAGCTAATTTCACCGTTGCCAAAACCGCCTGAAGAGAAGAAAGAAGAAGCCAAAGATCCACCCAAGGAAGAAGAGAAAAAAGATGAGGTCAGCAGTATTGGGCACGTTTTGCAAGGAAAGATAAAGAACATTTAG

amino acid sequence:

MGEEKKQEEKKEEAKEEEKKEEKKEEEPPEIVLKVDMHCEACARKVARALKGFEGVEQVSTDSKASKVVVKGKAADPSKVCERLQKKSGRKVELISPLPKPPEEKKEEAKDPPKEEEKKDEVSSIGHVLQGKIKNI

**11. PnCCH12**

nucleotide sequence:

ATGTTTCAATGGCGATTTGGGAGGTCAAAATTATCCAACGCCTTGTCTACTGTGGAGCTCCTGGTACATATGGATTGTGAAGGATGTGAAAAGAGAATACGAAGAGCAATCTCGAAAATTGATGGTGTTGATAGCTTGGAAATAGAAATGGATAAGCAAAAGGTGACTGTAAAAGGGTATGTCGACCAGAGAAAGGTCCTGAAGGTAGTGAGAAGAACAGGAAGAAGAGCAGAGTTTTGGCCATTTCCATATGACAGTGAATATTATCCATATGCATCCCAATACTTGGATGAAACTACGTACACGACATCGTATAATTATTACAGACATGGGTTCAATGAAAGTGTTCATGGATACTTCCCAGACCAAGCTTACTGCACCGTCCCTGATGATACAGTCCATCTTTTCAGTGATGACAATGTCCATGCATATTGTAGTATTATGTAA

amino acid sequence:

MFQWRFGRSKLSNALSTVELLVHMDCEGCEKRIRRAISKIDGVDSLEIEMDKQKVTVKGYVDQRKVLKVVRRTGRRAEFWPFPYDSEYYPYASQYLDETTYTTSYNYYRHGFNESVHGYFPDQAYCTVPDDTVHLFSDDNVHAYCSIM

**12. PnCCH15**

nucleotide sequence:

ATGGGTGCTCTTGACGATCTCTCAGATTACCTCTCAGACTTGTTTACAGCTGCCAGGAAGAAGAGGAAAAGAAAACCAATGCAGACAGTTGATATCAAAGTGAAGATGGATTGCGATGGCTGTGAAAGGAGGGTCAAAAATTCTGTTTCCTCCATGAAGGGCGTTAAATCAGTAGAAGTGAACAGAAAGCAAAGCCGGGTGACTGTCAGCGGGAATGTTGAGCCAAACAAGGTCTTGAAGAAAGTGAAGAGCACAGGAAAGAGGGCTGAGTTTTGGCCATATGTCCCATACAACTTGGTGGCTTATCCTTATGCTGCTCAAGCCTATGATAAGAAAGCACCTGCAGGCTATGTGAAGAATGTGGTTCAGGCACTTCCGAGCCCCAACGCAACAGATGAAAGATTTACATCTATGTTTAGCGATGAAAACCCGAATGCATGTTCCATCATGTAG

amino acid sequence:

MGALDDLSDYLSDLFTAARKKRKRKPMQTVDIKVKMDCDGCERRVKNSVSSMKGVKSVEVNRKQSRVTVSGNVEPNKVLKKVKSTGKRAEFWPYVPYNLVAYPYAAQAYDKKAPAGYVKNVVQALPSPNATDERFTSMFSDENPNACSIM

**13. PnCCH17**

nucleotide sequence:

ATGACTATCACAGAGATGAAAGTCTATATGGATTGTGCTGGCTGCGAGACCAAGATAAGGAAGGCTATTCAAAAACTAGATGGAGTGGATGATATCGATATAGACATATATATGCAAAAAGTAACAGTTATGGGATGGGCAGACCAGAGAAAAGTTCTTAAAGCAGTGAGGAAGACAGGAAGAAGAGCTGAGCTATGGCCATACCCATACAATCCTGAATCCTATAACTTCAACCAACAGTACTATTATCAGCAGCAGCATGAACAAGAAACAGTTACTTACTATGAAAAGAAGCCTACCGCTTCATACAACTACGACAAGCATGGCTACAATGAAGAAGAGTTTGGTTACTATCAAAAGCCAGCTTATGCCACCATTGTTGATGAAGAAGCTAGTGCCATCTTCAGTGATGAAAATCCTCATGCCTGCTCCATCATGTAA

amino acid sequence:

MTITEMKVYMDCAGCETKIRKAIQKLDGVDDIDIDIYMQKVTVMGWADQRKVLKAVRKTGRRAELWPYPYNPESYNFNQQYYYQQQHEQETVTYYEKKPTASYNYDKHGYNEEEFGYYQKPAYATIVDEEASAIFSDENPHACSIM

**14. PnCCH19**

nucleotide sequence:

ATGGCTACCTTCTTAGAGAGAGCATTTAAGACTATCATCTCTACCATCACTCACTCCTACTTTTACTTTCAAGAAGACCACGTAAGGATCAAAAATATCAGACACAACATGCGGAAGGGAAGACCACTTTCTTTGCAGACTGTTGAGCTCAAGGTCAGGATGTGCTGCGCTGGCTGTGAAAGGGTTGTCAAAAATGCCATTTATAAGCTTAGAGGTATTGATTCAGTGGAGGTTGACCTAGAGATGGAAAAGGTGACGGTGGTGGGGTATGTTGATCGCAACAAGGTCCTAAAGGCAGCGAGAAGGGCAGGAAAGAGGGCGGAATTCTGGCCATATCCGGATCTACCGCTGTACTTTACATCAGCAAATAACTATTTCAAGGACACAGCAAGTGAGTTCAAAGAGAGTTACAACTATTACAAGCATGGCTACAATCTTGCAGACAGGCACGGGACCATTCCGGTGAGCCACCGGGGAGATGACAAGGTCAGCAACATGTTCAATGATGACAATGTTAATGCCTGCTGTCTCATGTAG

amino acid sequence:

MATFLERAFKTIISTITHSYFYFQEDHVRIKNIRHNMRKGRPLSLQTVELKVRMCCAGCERVVKNAIYKLRGIDSVEVDLEMEKVTVVGYVDRNKVLKAARRAGKRAEFWPYPDLPLYFTSANNYFKDTASEFKESYNYYKHGYNLADRHGTIPVSHRGDDKVSNMFNDDNVNACCLM

**15. PnCCH20**

nucleotide sequence:

ATGGGAGCTCTTGATTACCTCTCCAACTTTTGCACTGTCACCAGCACAAGAAGCAAACGAAAACCAATGCAGACAGTTGAGATCAAGGTGAAGATGGACTGTGATGGCTGCGAAAGAAGAGTAAAAAATGCTGTCACCTCCATGAAAGGAGTAAAGACGGTGGAAGTGATCAGAAAACAAAGCAGGGTGGTGGTTAGTGGATATGTCGATCCAAACAAAGTCTTAAGAAGAGTGAAGAGCACAGGAAAGGTAGCTGAATTTTGGCCATATATCCCTCAACATCTAGTTTACTATCCTTATGTTTCCGGTGCCTATGACAAGAGGGCACCCGCGGGCTATGTTCGCAATGTTGTGCAAGCTTATCCAGCCTCAAATGCACCTGAAGACAACATTGTATCTCTCTTTAGTGATGATAATGTGAATGCTTGTTCCATCATGTAA

amino acid sequence:

MGALDYLSNFCTVTSTRSKRKPMQTVEIKVKMDCDGCERRVKNAVTSMKGVKTVEVIRKQSRVVVSGYVDPNKVLRRVKSTGKVAEFWPYIPQHLVYYPYVSGAYDKRAPAGYVRNVVQAYPASNAPEDNIVSLFSDDNVNACSIM
